# Knowledge, attitudes and practice of primary health care physicians towards hepatitis B virus in Al-Jouf province, Saudi Arabia

**DOI:** 10.1186/1756-0500-7-288

**Published:** 2014-05-09

**Authors:** Ahmad H Al-Hazmi

**Affiliations:** 1 Department of Family Medicine, College of Medicine, AlJouf University, King Khalid road, Al-Jouf, P.O. Box 2014, Sakaka 75741, Saudi Arabia

**Keywords:** Attitudes, Continuity of care, Hepatitis B, Knowledge, Primary health care, Saudi Arabia

## Abstract

**Background:**

Primary health care (PHC) physicians will be in the forefront of managing hepatitis B (HBV) patients. In Saudi Arabia, very little is known about knowledge, attitudes, and practice of PHC physicians towards HBV. This study aimed to assess the same parameters.

**Methods:**

During April 2012, a cross-sectional survey of 180 practitioners aged 38.1 ± 10.3 years was carried out in the primary health care centers (PHCCs) in AlJouf Province of Saudi Arabia. The physicians were asked to fill a valid questionnaire containing their sociodemographic data, and well-modified questions regarding their knowledge base, attitudes, and practice towards HBV. Data was processed and analyzed using SPSS (version 17) program, the level of significance was set at *P* < 0.05.

**Result:**

Response rate 88.3% yielded 159 questionnaires for analysis. Majority of the physicians surveyed 128 (80.6%) believed that PHC physicians are capable to achieve a major role in the management of HBV. 119 (74.8%) physicians surveyed were willing to manage HBV patients and 127 (79.9%) believed that vaccination is the most effective means to prevent HBV. There was a statistical significant correlation between physicians’ qualifications and continuity of care for HBV patients (32.8% vs 23.4%; *p* = 0.006), while continuality of care was more frequent among physicians with higher degrees compared to graduate physicians. Only 69 (43.4%) physicians were able to interpret HBV seromarkers. The vast majority of the physicians 142 (89.3%) were willing to subscribe in regular training programs about HBV.

**Conclusion:**

Suitable attitudes with lack of knowledge are found, and practice of our physicians with regard to this significant health issue appeared inappropriate. More education focusing on HBV is recommended.

## Background

Hepatitis B virus (HBV) represents important, yet clinically silent public health significance worldwide [1]. World health organization (WHO) data estimate serologic evidence of past or present HBV infection among two billion persons globally, while over 350 million people are carriers of chronic hepatitis B (CHB) worldwide [1,2]. The virus is transmitted in the blood and fluids which can be diagnosed during acute and chronic stages [3]. Acute infection may cause nonspecific symptoms or fulminant hepatitis that may die or requires urgent liver transplantation [3]. Chronic infection with HBV causes death associated with cirrhosis, liver failure, or hepatocellular carcinoma, and has a negative impact on health-related quality of life (HRQOL) [2-4]. HBV ranks second, after tobacco, on the list of known carcinogenic agents affecting humans and the tenth leading cause of death globally [5,6]. Costs associated with HBV are therefore substantial; it creates a significant economic burden to the health care services [1,4,7].

PHC physicians will be in the forefront of managing patients with chronic hepatitis B infection and most hepatitis B infections are identified at the primary care level [8,9]. It is assumed that physicians practicing in our community are lacking the important knowledge about and the appropriate attitudes toward HBV. Although, many studies in different countries have evaluated the knowledge level, attitudes, and practice among PHC physicians towards HBV [9-11]. There have been a very few attempts to identify a specific impact of this condition among physicians practicing in Saudi Arabia, a country with the highest high endemicity of HBV [12]. Hence, the main reasons of this study are to evaluate the knowledge base, attitudes, and to explore practice patterns of PHC physicians towards HBV. The data and information obtained from this study will identify weaknesses and help to improve healthcare system in managing this serious disease in the primary care setting.

## Methods

### Study design and setting

A cross-sectional descriptive study was carried out on physicians practicing at PHCCs in AlJouf province of Saudi Arabia (population 3.5 × 10^5^). The province contains four towns Domat alJanddal, Zallom, Sowair and Sakaka. There were around 200 physicians practicing in a total 45 PHCCs. Out of these 45, forty PHCCs were selected for the present study.

### Questionnaire

The well-structured questionnaire was developed to assess the KAP of PHC physicians regarding HBV. The study questions and the characteristics of participants were based on previous surveys and articles with similar objectives [7-11,13-15]. For validating the questionnaire, specific experts in standardizing questionnaire, gastroenterologists, hepatologists, community medicine and biostatistics, confirmed the introduction and validity contents of the primary questionnaire. Some questions were omitted according to their advice. The questionnaire comprised of 39 “closed ended questions”. The first part contained 33 with variable items (3–15) focusing on physicians’ knowledge, attitudes, and the most importantly, clinical points in the management of this condition [1-3,7-9]. The second part covered demographic characteristics of physicians (age, gender, graduation level, years in practice, type of job, and nationalities). The study protocol was discussed and approved by the research and ethical committee in AlJouf University College of Medicine.

During April 2012, 180 self-administered anonymous confidential questionnaires with explanatory letters were distributed to the PHC physicians practicing in 40 PHCCs. We started with meeting the director of the centre and giving a ten minutes introduction, explaining objectives of the study and going through all the questions with the physicians. The physicians were then asked to fill the questionnaires in their clinics and return them back to the investigator in same day.

On 15^th^ march 2012, the questionnaire was pre-tested during a pilot study that was conducted in 8 PHCCs inside Sakaka city. This was done to ensure clarity, relevance and to determine the amount of time needed to answer all items. The result of the pre-test was evaluated critically and some modifications were made accordingly. The average time needed to fill all items in the questionnaire was about 25 minutes. Results of the pilot study were not included in the final analysis.

### Statistical analysis

Data were analyzed using SPSS packed version 17.0 (IBM SPSS Inc., Chicago, IL, USA). *p* < 0.05 was considered significant; Chi-square test was applied for analysis of categorical data. Mean ± SD and proportions were used to describe continuous and dichotomous data, respectively.

## Results

Out of the 180 questionnaires distributed, 159 (response rate: 88.3%) were collected and analyzed. The general characteristics of physicians are shown in Table 1. A majority of the physicians were males 128 (80.5%), and the number of the female physicians was only 31(19.5%). The male to female ratio was found to be 4.1:1; with mean ± SD age were 38.1 ± 10.3 years (range 27 to 65 years). Physicians with ≥10 years of practice constituted less than half of the sample 76 (47.8%). Less than half 0f the physicians 59 (37.1%) had post graduate degrees, and only 9 (5.7%) were Saudi nationals. Of the physicians surveyed, 43 (27%) see an average of 3 HBV patients per week, 25 (15.7%) diagnose an average of less than 5 HBV patients per month. Whereas, 23 (14.5%) refer an average of 3 patients per month.

**Table 1:** Distribution of primary health care physicians according to their general characteristics

**Characteristics**	**Primary health care physicians ( N = 159)**	
	**No**	**%**
Gender:		
■ Male	128	80.5
■ Female	31	19.5
Type of job		
■ Governmental	129	81.1
■ Private	30	18.9
Age		
■ < 30 years	10	6.3
■ 30-40 years	82	51.6
■ 41-50 years	38	23.9
■ 51-60 years	29	18.2
Post-graduate Qualifications:		
■ MBBS	96	60.4
■ Diploma	23	14.5
■ Master Degree	29	18.2
■ Board or PhD	11	6.9
Years in practice:		
■ ≤ 5 years	17	10.7
■ >5-10 years	65	40.9
■ >10-20 years	53	33.3
■ >20-30 years	21	13.2
■ >30 years	3	1.9
Nationalities:		
■ Saudi	9	5.7
■ Egyptians	65	40.9
■ Sudanese	24	15.1
■ Syrian	24	15.1
■ Jordanian	4	2.5
■ Indian	8	5.0
■ Pakistani	8	5.0
■ Bangladeshi	9	5.7
■ Other nationalities	8	5.0

When asked about what would they do if a patient with HBV presents to their office, we found only 30.8% (n = 45) of the physicians responded that they will achieve continuity of care for HBV; whereas 62.9% (n = 100) of the participants will refer HBV immediately or later to the specialists. Four males and three female physicians (4.4%) were not sure what should be done for HBV.

When inquired about whether the PHC physicians are capable to achieve a major role in the management of HBV; 85 (53.5%) agreed, 43 (27.1%) strongly agreed, 13 (8.2%) disagreed, 4 (2.6%) strongly disagreed, and 12 (7.7%) were not sure.

Table 2 presents physicians’ correct answer rates on knowledge about HBV distributed by their qualifications. It is obvious that in each area the mean proportion of the correct answers varied. Overall, the mean knowledge level among the physicians surveyed was 62.9%, (range 28.9 to 88.1%). The maximal ranking was pertaining to the physicians’ knowledge about an acute infection may cause nonspecific symptoms and the minimal ranking was pertaining to their knowledge about the number of HBV serotypes. There were no statistical significant correlations between physicians’ qualifications and their knowledge about HBV in all items.

**Table 2:** Physicians’ knowledge about HBV in relation to their qualifications (correct answer rates)

**Question item: (correct answer) ‡**	**Correct; n = 159 (%)**	**PHC physicians’ qualifications**				**X**^**2**^	**P value**
		**MBBS; (n = 96)**	**Diploma, (n = 23)**	**Masters degree, (n = 29)**	**Board or PhD (n = 11)**		
Acute infection may cause nonspecific symptoms; (T)	140 (88.1)	89 (92.7)	19 (82.6)	24 (82.8)	8 (72.7)	7.126	0.129
Chronic infection is characterized by persistence of HBsAg (with or without concurrent HBeAg); (>6 months).	96 (60.4)	60 (62.5)	13 (56.5)	18 (62.1)	5 (45.5)	14.577	0.556
Chronic infection will develop in almost all children infected perinatally; (T)	64 (40.3)	39 (40.6)	8 (34.8)	13 (44.8)	4 (36.4)	9.139	0.331
Transmission after needlestick is higher for HIV in comparison with HBV; (F)	89 (56.0)	54 (56.3)	9 (39.1)	20 (69.0)	6 (54.5)	8.711	0.367
Seven genotypes of HBV have been identified labeled A through G; (F)	46 (28.9)	22 (22.9)	9 (39.1)	10 (34.5)	5 (45.5)	15.618	0.209
Mothers who are both HBsAg and hepatitis B “e” antigen (HBeAg)-positive pass the virus perinatally to 70% to 90% of their offspring; (T)	123 (77.4)	72 (75.0)	18 (78.3)	24 (82.8)	9 (81.8)	5.085	0.748
Treatment for chronic viral hepatitis B is very expensive and not always effective; (T)	119 (74.8)	71 (74.0)	14 ( 58.3)	24 (82.8)	10 (90.9)	10.275	0.246
The most effective means to prevent HBV infection is through vaccination; (T)	127 (79.9)	77 (80.2)	18 (78.3)	24 (82.8)	8 (72.7)	5.812	0.668
HBV replicates only hepatocytes; (T)	97 (61.0)	54 (56.3)	15 (65.2)	21 (72.4)	7 (63.6)	9.117	0.693

‡ Some physicians did not respond to some items in some questions; *HBV*- Hepatitis B virus; *T* = true, *F* = false.

Table 3 shows physicians’ attitudes toward HBV. The study has shown that, 106 (66.7%) physicians did not have concern on shaking hands with a person who has HBV and 108 (67.9%) physicians did not feel uncomfortable hugging him. Having a person with CHB in the same workplace was accepted by only 62 (39.0%) physicians. The vast majority 139 (85.5%) were willing to get more training programs about HBV and suggested to establish “Saudi guidelines to care and manage HBV patients”.

**Table 3:** Primary health care physicians’ attitudes toward HBV (N = 159)

**Attitudinal item**	**Strongly disagree; N (%)**	**Disagree; N (%)**	**Neutral; N (%)**	**Agree; N (%)**	**Strongly agree; N (%)**
I have concern to get HBV from my patients	19 (11.9)	30 (19.0)	30 (19.0)	67 (42.1)	13 (8.2)
I would not mind having a person infected with HBV in my working place	22 (13.8)	42 (26.4)	33 (20.8)	49 (30.8)	12 (7.6)
I have concern in shaking hands of persons infected with HBV infection	46 (28.9)	60 (37.7)	27 (17.0)	16 (10.1)	9 (5.7)
I would feel uncomfortable hugging a person who has HBV	38 (23.9)	70 (44.1)	17 (10.7)	29 (18.2)	5 (3.1)
Chronic infection with HBV is shameful	65 (40.9)	54 (34.0)	23 (14.5)	13 (8.2)	3 (1.9)
It is difficult to eradicate HBV	12 (7.6)	42 (26.4)	35 (22.0)	53 (33.3)	17 (10.7)
More attention should be offered to HBV	15 (9.4)	5 (3.2)	7 (4.4)	72 (45.3)	60 (37.7)
I wish to get more training programs about HBV	10 (6.3)	2 (1.3)	10 (6.3)	59 (37.1)	76 (48.0)
Can participate in all activities including contact sports	6 (3.8)	23 (14.5)	28 (17.6)	74 (46.5)	26 (16.4)

Table 4 shows physicians’ responses on different aspects of practice toward HBV. By the findings it is revealed that 119 (74.8%) physicians would take care of HBV patient. Most of the physicians (n = 139; 87.4%) were willing to collaborate with other health professionals, especially trained nurses, dieticians, and hepatologists on management of HBV patients. Of all the physicians surveyed, only 7 (4.4%) prescribe some forms of complimentary medications for HBV patients.

**Table 4:** Practices of primary health care physicians towards HBV: Options in the management of patients with HBV include the following as needed

**In your clinic; options in care of patients with HBV include the following as needed: ‡**	**Do N (%)**	**Don’t N (%)**	**Don’t know N (%)**
I would take care of an infected-person with HBV	119 (74.8)	24 (15.1)	14 (8.8)
Persons with chronic HBV infection should be monitored for disease activity	142 (89.3)	7 (4.5)	7 (4.5 )
Sexual partners should be immunized	145 ( 91.0)	11(7.1 )	3 (1.9)
Involvement of the family in education of persons infected with HBV	143 (90.0)	10 (6.3)	6 (3.8)
All medications should be taken under physician instructions	146 (91.8)	7 (4.5)	6 (3.8)
Irregular food regimens can worsen HBV infection	79 (49.7)	53 (33.3)	27 (17.1)
Not share toothbrushes or razors	141 (88.7)	11 (7.1 )	7 (4.5)
Persons with chronic HBV infection who are not immune to hepatitis A should receive two doses of hepatitis A vaccine at least six months apart	91 (57.2)	30 (18.9)	38 (23.9)
If you are traveling to a country where hepatitis B is common, try to get all the shots before you go	136 (85.5)	17 (10.7)	6 (3.8)
Health care professionals should receive hepatitis B vaccination	141 (88.7)	7 (4.5)	6 (3.8)
Encourage the family members and other close personal contacts to get tested	147 (92.3 )	4 (2.5)	7 (4.5)
Collaborations with other health professionals, especially trained nurses, dietitians, and hepatologists are very important tools for care of patients with HBV.	140 (88.1 )	7 (4.5)	12 (7.6)
I feel confident in dealing with patient who is HBsAg positive	84 (52.8)	41 (25.8)	34 (21.4)

‡ Some physicians did not respond to all items; *HBV*- Hepatitis B virus.

Table 5 shows physicians’ knowledge on HBV vaccine distributed by their years in practice. Only 63 (39.6%) physicians surveyed recognized that the duration of protection is at least 15 years after receiving 3 doses of HBV vaccine and, based on current scientific evidence, lifelong. Also, 80 (50.3%) of the sample regarded pregnancy is contraindication for use of this vaccine.

**Table 5:** Knowledge of PHC physicians regarding HBV vaccine in relation to their years in practice

**Question item regarding HBV vaccine ‡**	**Correct; n = 159 (%)**	**Physicians’ years in practice**			**X**^**2**^	***P *** **value**
		**<10 yrs. N = 81 (%)**	**10-20 yrs. N = 52 (%)**	**>20 yrs. N = 24 (%)**		
Is safe for people of all ages; (T)	130 (81.8)	63 (77.8)	45 (86.5)	22 (91.7)	4.549	0.919
Hepatitis B vaccine and other vaccines administered during the same visit should be given at different injection sites; (T)	119 (74.8)	63 (77.8)	40 (76.9)	16 (66.7)	8.706	0.368
The duration of protection is at least 15 years and, based on current scientific evidence, lifelong; (T)	63 (39.6)	32 (39.5)	24 (46.2)	7 (29.2)	4.730	0.316
The complete vaccine series induces protective antibody levels in >95% of infants, children and young adults; (T)	115 (72.3)	50 (61.7)	44 (84.6)	21 (87.5)	10.137	0.035
Hepatitis B vaccine can be given safely together with any other vaccine, and vice versa; (T)	117 (73.6)	60 (74.1)	39 (75)	18 (75)	5.838	0.442
Pregnancy is a contraindication for use of this vaccine; (F)	80 (50.3)	36 (44.4)	30 (57.7)	14 (58.3)	6.699	0.568

‡ *HPV* = Hepatitis B virus, *T* = true, *F* = false.

Regarding the possible modes for HBV transmission, 142 (89.3%) physicians reported through wound-to-wound, 140 (88.1%) sexual contact, 127 (79.9%) during delivery from the infected mothers to their infants, 110 (69.2%) maternofetal, 83 (52.2%) through saliva followed by 81 (50.9%) breast milk. While, 130 (81.8%) physicians considered hugging, 136 (85.5%) shaking hands or 123 (77.4%) sharing foods with infected persons as a safe practices.

When asked about the main sources of their knowledge about HBV; textbooks were ranked first among 139 (85.5%) physicians, followed by internet 15 (9.4%), whereas medical journals were the main sources among 8 (5.0%) physicians.

## Discussion and conclusion

Hepatitis B virus is a challenging infectious disease for all of the health care professionals throughout the world [1-3]. Fortunately, infection with this virus is not only treatable, but also preventable [2-5]. PHC physicians are, thus, expected to achieve a major role in the diagnosis, prevention, and referral of HBV, as a means of controlling HBV [7-11]. The current study is, to the best of the author’s knowledge, the first comprehensive PHC setting-based survey carried out in Saudi Arabia (alJouf province) to evaluate knowledge, attitudes and practice of PHC physicians regarding management of HBV in the PHCCs. We believe that our sample was representative of the PHC physicians and accounted for approximately 80% of all the PHC physicians practicing in alJouf province. More than half the physicians under this study had low to moderate experience in PHC setting (≤10 years).

This study, clearly, proves that physicians practicing in our community were aware of magnitude of HBV in Saudi Arabia [12]. It is found, most of the physicians 102 (64.2%) perceived that HBV at level of public health significance in the country; and 131 (82.4%) suggested the need of catch-up vaccination for all Saudi older age groups, including adolescents and adults. Also, 143 (89.9%) suggested “educational strategies about HBV with special attention to Saudi citizens of lower education levels and lower socio-economic classes”. Our findings are in agreement with, and support, recommendations and reports of CDC about HBV [13]. The referred recommendations and reports demonstrate that more actions should be offered to HBV in the highly endemic areas like Saudi Arabia where HBV prevalence is ≥8% [12,13].

It is well documented that most patients with chronic HBV are identified in the primary care level and only those with chronic active HBV are selected for specialist care [9,10,14]. Unfortunately, we found only 45 (28.3%) physicians surveyed will carry a continuity of care for HBV patients; whereas, 99 (62.3%) will refer HBV to the specialists. However, an effective referral is needed only for those who are eligible for sophisticated diagnosis and antiviral therapy [9,10,14]. Our findings are in agreement with those practical difficulties in the management of HBV in Asia-Pacific [7]. The referred reports have demonstrated that PHC physicians may show a reluctance to treat HBV patients, because of incomplete knowledge about hepatitis B, resulting in the referral of numerous patients to specialists [7]. Also, lack of suitable approach or confidence with lack of diagnosis facilities all contribute to referral [7,11].

This study shows inadequate knowledge among PHC physicians regarding HBV, but this not necessarily reflects the picture of PHC physicians who are practicing in other parts of Saudi Arabia. This study has shown that, only 69 (43.4%) physicians were able to interpret HBV seromarkers and only 67 (42.1%) recognized the incubation period of HBV [13]. Also, 97 (61%) of the physicians were unaware of the risk of HBV chronicity which is inversely related to age [2,3,13]. Attributable reasons could be inadequate training programs about HBV. Our findings are not dissimilar to those reported in a recently study carried out in Australia to evaluate the knowledge and educational needs of GPs about viral hepatitis [15]. The referred study has identified limitations in knowledge among GPs concerning certain aspects of viral hepatitis [15].

Health education is of crucial significance for persons with HBV [6-9,11,13]. Those individuals should be educated about the risk of transmission, needle exchange programs, condom use, and avoidance of sharing toothbrushes, razors, etc. [11,13]. It was interesting to find most of the physicians 142 (89.3%) had interest to involve the family on education and management of HBV patients. As such, educational skills about HBV could optimize care and management of patients and increase the doctor-patient relationship [1,4,6,7].

Hepatitis B is one of the most highly infectious diseases without seasonal distribution [6]. Our findings show a high rate of misconceptions regarding infectivity patterns of HBV. It was found, only 37 (23.3%) physicians recognized that HBV could be an infectious agent in the environment and more than 45% were not aware of its transmission through needlestick which is 50–100 times higher in comparison with HIV [2,3,6,13]. Misconceptions and confusion that persist among PHC physicians in these aspects could interfere with patients’ education and the safety of household contacts [2,6,13]. The possible explanation could be that, during an undergraduate study the infectious diseases, such as HBV are given only a small share in comparison to other medical subjects. In addition, textbooks were the main source of knowledge among the physicians surveyed and most textbooks are not updated, all of these issues could contribute to a lack of knowledge about HBV.

The most effective method of preventing HBV is through vaccination [2,4-6,13]. Interestingly, the vast majority of the physicians believed that vaccination is the mainstay to address HBV, and more than 81% of them recognized that the vaccine is safe for people of all ages [2,6,13]. Our cross-sectional study has shown adequate knowledge among PHC physicians regarding modes of HBV transmission. For specific modes of transmission (for example hugging, sharing food, maternofetal, breast milk) there were misconceptions [2-4,13]. This might be due to the controversy of these issues and the lack of the solid evidence. It is observed that maternofetal transmission is the dominant route of HBV transmission in high prevalence endemic areas such as South-East Asia [16]. Breast-feeding seems to be an additional mode by which infants acquire HBV; however, the risk associated with breast-feeding is negligible compared with that of exposure to maternal blood and body fluids at birth [6,16,17].

In the area of follow-up, we found 100 (62.9%) physicians suggested it is more appropriate for CHB patients with [HBeAg-ve, liver enzymes within normal limits] to be followed in the primary care. Whereas, nearly all the participants 150 (94.3%) reported it is more appropriate for those with (HBeAg + ve, elevated liver enzymes) to be referred to the specialists. Our findings are in agreement with several international strategies regarding prevention, care, and follow-up of HBV [18,19]. The referred strategies have demonstrated that patients in inactive carrier stage do not need treatment, because the progression of their liver disease is slow, if at all [18,19]. Only those patients who have CHB (active HBV replication with high viral load and ongoing necro-inflammation) qualify for treatment [18,19].

This study has revealed that only minority of the PHC physicians 25 (15.7%) considered that, PHCCs are a suitable places to manage HBV patients. Our findings and those internationally revealed that lack of investigation facilities in the PHCCs contribute further to poor management of HBV patients in the PHCCs [11]. It was found, the proportion of private physicians who considered PHCCs suitable places to manage HBV is more than that found among those governmental administered physicians (25.0% vs 17.0%; p < 0.05). Attributable reasons could be adequate facilities at the private health care centers in comparison with that available in public PHCCs [11].

This study shows more suitable attitudes among PHC physicians toward HBV. It was found, 101 (63.7%) physicians reported that, persons with HBV can participate in all activities including contact sports, and 119 (75.1%) believed that they should not be excluded from the daycare or school participation and should not be isolated from other children. Also, 106 (66.7%) physicians did not have concern on shaking hands and did not feel uncomfortable hugging a person who has HBV. Our findings are in agreement with those internationally which demonstrate that no evidence exists of HBV transmission by causal contact in the workplace, and HBV is not spread by kissing or hugging [13].

This study has revealed that the vast majority of the physicians were in favour to establish “Saudi guidelines for diagnosis and management of hepatitis B virus”. This finding is in agreement with, and supports, those recommendations regarding management of CHB in resource-poor countries. The referred recommendations revealed that guidelines for primary care physicians should be published and existing ones should be updated to keep physicians informed on changes in CHB management [1].

Strengths of this study including a high response rate (88.3%), also, targeting PHC physicians that have been involved as a “gatekeepers” to the primary protective and curative health services, moreover, to the best of our knowledge, no similar study has been carried out among PHC physicians in Saudi Arabia. Despite of the study findings, we acknowledge its limitations, actually all the information are reported which may not be reflective of the actual situation in the primary care, also, all the questions in the questionnaire are close-ended which may hinder some important points on knowledge and practice of the participating physicians.

In conclusion, suitable attitudes with lack of knowledge were found and practice of PHC physicians regarding HBV appears inappropriate among the majority of PHC physicians in Al Jouf province of Saudi Arabia. This may be due to lack of structured training programs concerning HBV. A well planned CME programs must be conducted to increase their knowledge and correct erroneous ideas regarding HBV. PHCCs should be technically supported to facilitate the diagnosis of HBV. Further studies are also required to identify other factors underlining the less than optimal preventive and management of HBV in the primary care setting.

## Competing interests

There is none to declare.
